# A Framework for the Optimization of Complex Cyber-Physical Systems via Directed Acyclic Graph

**DOI:** 10.3390/s22041490

**Published:** 2022-02-15

**Authors:** Manuel Castejón-Limas, Laura Fernández-Robles, Héctor Alaiz-Moretón, Jaime Cifuentes-Rodriguez, Camino Fernández-Llamas

**Affiliations:** Department of Mechanical, Computer Science and Aerospace Engineering, Universidad de León, 24071 León, Spain; manuel.castejon@unileon.es (M.C.-L.); hector.moreton@unileon.es (H.A.-M.); jcifr@unileon.es (J.C.-R.); camino.fernandez@unileon.es (C.F.-L.)

**Keywords:** Cyber-Physical Systems, Lean Manufacturing, Directed Acyclic Graphs, scikit-learn, pipegraph, machine learning models

## Abstract

Mathematical modeling and data-driven methodologies are frequently required to optimize industrial processes in the context of Cyber-Physical Systems (CPS). This paper introduces the PipeGraph software library, an open-source python toolbox for easing the creation of machine learning models by using Directed Acyclic Graph (DAG)-like implementations that can be used for CPS. scikit-learn’s Pipeline is a very useful tool to bind a sequence of transformers and a final estimator in a single unit capable of working itself as an estimator. It sequentially assembles several steps that can be cross-validated together while setting different parameters. Steps encapsulation secures the experiment from data leakage during the training phase. The scientific goal of PipeGraph is to extend the concept of Pipeline by using a graph structure that can handle scikit-learn’s objects in DAG layouts. It allows performing diverse operations, instead of only transformations, following the topological ordering of the steps in the graph; it provides access to all the data generated along the intermediate steps; and it is compatible with GridSearchCV function to tune the hyperparameters of the steps. It is also not limited to (X,y) entries. Moreover, it has been proposed as part of the scikit-learn-contrib supported project, and is fully compatible with scikit-learn. Documentation and unitary tests are publicly available together with the source code. Two case studies are analyzed in which PipeGraph proves to be essential in improving CPS modeling and optimization: the first is about the optimization of a heat exchange management system, and the second deals with the detection of anomalies in manufacturing processes.

## 1. Introduction

Continuous technological advancements in fields such as Information Technology (IT), Artificial Intelligence (AI), and the Internet of Things (IoT), among others, have drastically transformed manufacturing processes. Recent technological advancements have permitted a systematic deployment of Cyber–Physical Systems (CPS) in manufacturing, which allows intertwining physical and software components to control a mechanism by means of a computer system. CPS has considerably improved the efficiency of production processes while also making them more resilient and collaborative [1]. These cutting-edge technologies are advancing the manufacturing economic sector in the Industry 4.0 era [2].

In the Industry 4.0 paradigm, manufacturing industries must modify their management systems and look for new manufacturing strategies [3,4] to find solutions to tackle the issues faced nowadays. Lean Manufacturing (LM) has become one of the most generally accepted manufacturing methods and management styles used by organizations throughout the world to improve their business performance and competitiveness [5]. Since LM improves operational performance for manufacturing organizations in developing and developed countries [6], it has spread all over the world [4]. Ref. [7] suggested that the future research methodologies for LM can be classified into meaningful themes, namely: the size of the research sample and its composition; several types of study (other than surveys); longitudinal studies; applying advanced statistical analysis and (mathematical) modeling techniques; objective, real and quantitative data; surveys; mixed/multiple research studies; reliability and validity analysis; using computer-aided technology for data collection, and processing and research collaborations.

This paper focuses on the application of mathematical modeling techniques and the use of computer-aided technology for data processing for LM CPS, at the core of Industry 4.0. LM deals with the optimization of the performance according to a specific set of principles [8]. In the context of CPS, mathematical modeling and data-driven techniques are usually needed to optimize industrial processes [9]. Ref. [10] presented a fully model-driven technique based on MontiArc models of the architecture of the CPS and UML/P class diagrams to construct the digital twin information system in CPS. Ref. [11] combined Random Forest (RF) with Bayesian optimization for large-scale dimension data quality prediction, selecting critical production aspects based on information gain, and then using sensitivity analysis to preserve product quality, which may provide management insights and operational guidance for predicting and controlling product quality in the real-world process industry. In [12], in the context of varying design uncertainty of CPS, the feasibility of appropriate evolutionary and Machine Learning (ML) techniques was examined. In [13], the Neural Network Verification (NNV), a software tool that offers a set of reachability methods for verifying and testing the safety (and robustness) of real-world Deep Neural Networks (DNNs) and learning-enabled CPS, was introduced.

ML is contributory in solving difficult problems in the domains of data-driven forecasting, classification and clustering for CPS. However, the literature of the ML field presents a high number of approaches and variations which makes difficult to establish a clear classification scheme for its algorithms [14]. Toolkits for ML aim at standardizing interfaces to ease the use of ML algorithms in different programming languages as R [15], Apache Spark [16], JAVA and C# [17], C++ [18,19], JAVA [20,21], PERL [22], JavaScript [23], command-line [24], among others. Python is one of the most popular and widely-used software systems for statistics, data mining, and ML, and scikit-learn [25] is the most widely used module for implementing a wide range of state-of-the-art ML algorithms for medium-scale supervised and unsupervised problems.

Data-driven CPS case studies usually need to split the data into training and test sets and to combine a set of processes to be applied separately to the training and test data. Some bad practices in data manipulation can end up in a misleading interpretation of the achieved results. The use of tools that allow the selection of the pertinent steps in an ad-hoc designed pipeline helps to reduce programming errors [26]. The Pipeline object of the scikit-learn module allows combining several transformers and an estimator to create a combined estimator [25]. This object behaves as a standard estimator, and GridSearchCV therefore can be used to tune the parameters of all steps. Nevertheless, Pipeline has some shortcomings such as: it is quite rigid since it strictly allows combining transformers and an estimator sequentially in such a way that the inputs of a step are the transformed outputs of the previous step; it requires (X,y)-like entries, the *X* is transformed by the transformers, and (X,y) is used by the estimator; GridSearchCV on a Pipeline is constrained to only split (X,y) and tune parameters that are variables of the functions, for example, a fit_param cannot be tuned.

**Figure 1:**
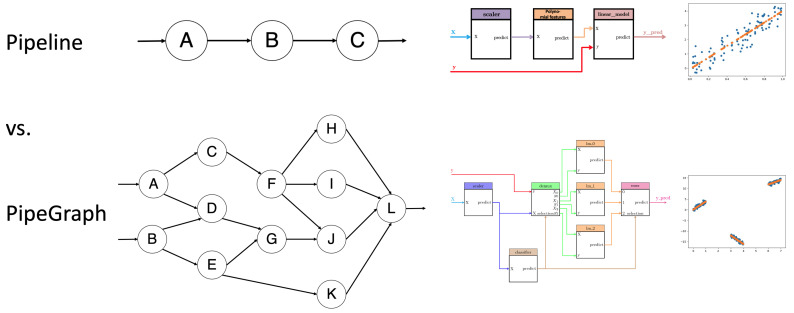
Graphical abstract. In the upper part, the Pipeline structure can be seen, which only allows sequential steps. At the bottom, the PipeGraph structure is shown. The combination of steps based on a directed acyclic graph makes a wide variety of operations feasible.

In this work we present PipeGraph, a new toolbox that aims at overcoming some of the weaknesses of Pipeline while providing greater functionality to ML users. In PipeGraph, all kinds of steps, not only transformers and an estimator, can be combined in the form of a Directed Acyclic Graph (DAG), the entries of a step can come from the outputs of any previous step or the inputs to the graph, see Figure 1. For more information about the application of DAGs in ML see [27]. A PipeGraph accepts more variables than (X,y) as inputs that can be appended or split within the graph to make use of the standard scikit-learn functions. This allows using hyperparameter tuning techniques such as GridSearchCV to easily manage data further than (X,y) inputs and tune other parameters apart from the variables of functions. The user can easily implement a new PipeGraph creating steps that make use of: (i) scikit-learn functions, (ii) provided custom blocks that implement basic functions, or (iii) their own elaborated custom blocks that implement custom functions. Moreover, in this paper we report two case studies where PipeGraph was essential to ease the modeling and optimization in the CPS area. The first case study deals with an optimization of heat exchange management system, whereas the second one deals with anomaly detection in manufacturing processes.

## 2. Data Leakage in ML Experiments

This section emphasizes the importance of avoiding data leakage in data driven numerical modeling. Data leakage in experimental learning is an important design defect that practitioners must consciously avoid. Best practices in ML projects establish that the information related to the case study must be split in a number of different data sets, i.e., training set, test set, and if necessary validation test [28]. This allows the practitioner to obtain a measure of the error expected on data unseen during the training process. It is crucial for the model to be evaluated on unseen data in order to confirm the generalization capability of the model. Moreover, cross–validation (CV) is one of the most popular strategies to obtain a representative value of the error measure. CV pursues to provide an error measure on unseen data by using the training set alone. To achieve that goal, it splits the training data set in a number *k* of subsets, also known as ‘folds’, and runs *k* training experiments by isolating one of those folds at a time, to later on measure the error on the specific set that has been isolated. Thus, in each of the *k* experiments the model is trained and tested on different sets. Finally, the error measures are condensed by using standard statistics like mean value and standard deviation.

One of the most important risks associated to CV is data leakage, by failing to correctly manage the different sets of data and their effect on the different stages of the training phase. Let us consider the following simple illustrative case: a linear model that is fitted using scaled data. Such an example requires the data to run through two processes: a scaler and then a predictive model. During the training phase the scaler annotates information related to the presented data, e.g., maximum and minimum, or mean value and standard deviation. This annotations allow the scaler process to eventually apply the same transformation to new and unseen data. A typical error that might occur is that the code for the CV presents the whole training data set to the scaler and then loops different fit experiments using the *k*-fold strategy. By doing so, each of the *k* models fails to provide an error measure that is representative of the behavior of the model on unseen data, because indeed, what is meant to be considered as unseen data has effectively polluted the experiment by its potential impact on the parameters annotated on the scaler.

One of the main strategies to avoid the risk of allowing the user to mishandle the flow of information during CV is to embed the sequence of processes in an entity that is handled in the code as a single unit. A remarkable example of such solution is the Pipeline class provided by scikit-learn. According to scikit-learn documentation “*pipelines help avoid leaking statistics from your test data into the trained model in cross-validation blue (CV), by ensuring that the same samples are used to train the transformers and predictors*” [25].

In the former example, the scaler cannot be trained using the whole data set during the CV experiment because Pipeline is in charge of training the scaler as many times as the predictive model. This is a very successful strategy for migrating the responsibility of orchestrating the fit and predict phases from the user to the code of the framework, thus avoiding possible user errors while handling the data.

CV is one of the scenarios where data leakage can occur, other notable situations prone to this design defect can occur when augmenting the data or during the feature engineering stage, to name a few.

PipeGraph is another example of such encapsulation. As Pipeline, it provides the researcher with the safety that no data leakage will occur during the training phase. Moreover, it enhances the capabilities of the standard Pipeline provided by scikit-learn by allowing non–linear data flows, as we will show in the following section.

Thus the scientific goal of this paper is to present researchers from the ML community, in particular those in the CPS area, a novel framework aimed at providing expressive means to design complex models such as those typically present in CPS. PipeGraph thus combines the expressive power of DAGs with the intrinsic safety of encapsulation.

## 3. Library Design

### 3.1. Project Management

*Quality assurance.* In order to ensure code quality and consistency, unitary tests are provided. We granted a coverage of 89% for the release 0.0.15 of the PipeGraph toolbox. New contributions are automatically checked through a Continuous Integration (CI) system for the sake of determining metrics concerning code quality.

*Continuous integration.* In order to ensure CI when using and contributing to PipeGraph toolbox, Travis CI is used to integrate new code and provide back-compatibility. Circle CI is used to build new documentation along with examples containing calculations.

*Community-based development.*PipeGraph toolbox is fully developed under GitHub and gitter to facilitate collaborative programming, this is, issue tracking, code integration, and idea deliberations.

*Documentation.* We provide consistent Application Programming Interface (API) documentation and gallery examples (https://mcasl.github.io/PipeGraph/api.html, accessed on 20 December 2021) by means of sphinx and numpydoc. A user’s guide together with a link to the API reference and examples are provided and centralized in GitHub (https://github.com/mcasl/PipeGraph, accessed on 20 December 2021).

*Project relevance.* At edition time of this paper PipeGraph toolbox has been proposed as part of the scikit-learn-contrib supported project.

### 3.2. Implementation Details

Here we describe the main issues that had to be solved for PipeGraph to work. First, scikit-learn step eligible classes provide a set of methods with different names for essentially the same purpose; providing the output corresponding to an input dataset. Depending on whether the class is a transformer or an estimator, this method can be called either transform, predict, or even fit_predict. This issue was originally solved by using the wrapper design pattern, although it has been proposed by scikit-learn core developers to consider the usage of mixin classes to provide a similar functionality. The development branch of the package already implements this alternative approach.

The main difference from a users perspective between using a Pipeline object or a PipeGraph object is the need of defining a dictionary that establishes the connections. Again, a scikit-learn core developer suggested the implementation of an inject method for that purpose and that approach is also already available along with the optional dictionary for those cases in which the user finds it more convenient to use.

The proposed toolbox depends on numpy, pandas, networkx, inspect, logging and scikit-learn and is distributed under MIT license.

PipeGraph can be easily installed using pip install pipegraph.

### 3.3. Example

We describe here one example for illustrative purposes. The system displays a predictive model in which a classifier provides the information to a demultiplexer to separate the dataset samples according to their corresponding class. After that, a different regression model is fitted for each class. Thus, the system contains the following steps:

scaler: A scikit-learn MinMaxScaler data preprocessor in charge of scaling the dataset.
classifier: A scikit-learn GaussianMixture classifier in charge of performing the clustering of the dataset and the classification of any new sample.
demux: A custom Demultiplexer class in charge of splitting the input arrays accordingly to the selection input vector. This block is provided by PipeGraph.
lm_0, lm_1, lm_2: A set of scikit-learn LinearRegression objects
mux: A custom Multiplexer class in charge of combining different input arrays into a single one accordingly to the selection input vector. This block is provided by PipeGraph.

**Figure 2:**
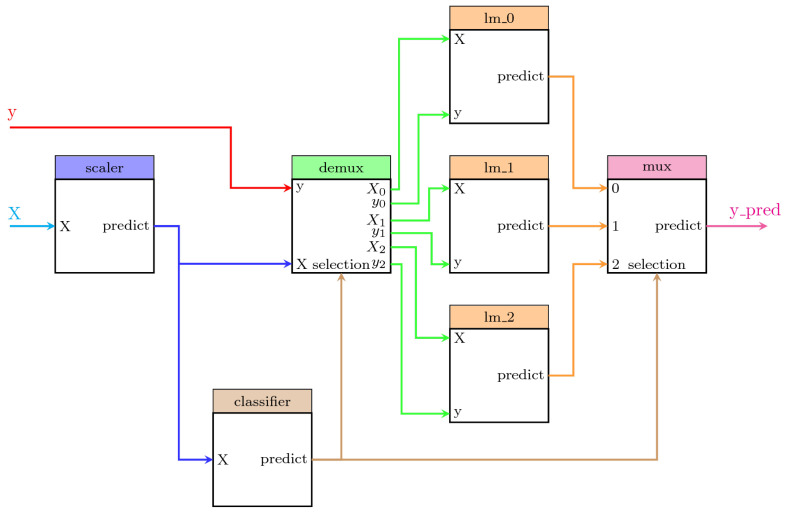
A PipeGraph system for fitting a different model per cluster.

This PipeGraph model is shown in Figure 2. It can be clearly seen in the figure the non sequential nature of such a system that cannot be otherwise described as a standard scikit-learn Pipeline.

The code for creating an artificial dataset and configuring the system is described in Listing A1 of Appendix A.

### 3.4. Implemented Methods

PipeGraph toolbox provides two interfaces, PipeGraphRegressor and PipeGraphClassifier, which are compatible with GridSearchCV and heavily based on scikit-learn’s Pipeline on purpose, as its aim is to offer an interface as similar to Pipeline as possible. By default, PipeGraphRegressor uses the regressor default score (the coefficient of determination R^2^ of the prediction) and PipeGraphClassifier uses the classifier default score (the mean accuracy of the prediction on given test data with respect to labels). As for the rest, both interfaces are equivalent.

The following functions can be used by the user in both interfaces:

- inject(sink, sink_var, source, source_var) Defines a connection between two nodes of the graph declaring which variable (source_var) from the origin node (source) is passed to the destination node (sink) with new variable name sink_name).
- decision_function(X) Applies PipeGraphClasifier’s predict method and returns the decision_function output of the final estimator.
- fit(X, y=None, **fit_params) Fits the PipeGraph steps one after the other and following the topological order of the graph defined by the connections attribute.
- fit_predict(X, y=None, **fit_params) Applies predict of a PipeGraph to the data following the topological order of the graph, followed by the fit_predict method of the final step in the PipeGraph. Valid only if the final step implements fit_predict.
- get_params(deep=True) Gets parameters for an estimator.
- predict(X) Predicts the PipeGraph steps one after the other and following the topological order defined by the alternative_connections attribute, in case it is not *None*, or the connections attribute otherwise.
- predict_log_proba(X) Applies PipeGraphRegressor’s predict method and returns the predict_log_proba output of the final estimator.
- predict_proba(X) Applies PipeGraphClassifier’s predict method and returns the predict_proba output of the final estimator.
- score(X, y=None, sample_weight=None) Applies PipeGraphRegressor’s predict method and returns the score output of the final estimator.
- set_params(**kwargs) Sets the parameters of this estimator. Valid parameter keys can be listed with get_params().

## 4. Case Studies

### 4.1. Anomaly Detection in Manufacturing Processes

The first case study deals with anomaly detection of machined workpieces using a computer vision system [29]. In that paper, a set of four classifiers were tested in order to choose the best model for identifying the presence of wear along the workpiece surface. Following a workflow consisting of a preprocessing phase followed by feature extraction and finally a classification step, the authors reported satisfactory results. In such scenario, the results of a deeper analysis could have been provided, where the quality of the classifier could have been improved by unleashing an additional parameter, the number of classes. Instead of assuming that two classes are present in the dataset, namely correct pieces and unacceptable pieces, according to the finishing quality, the result from considering more classes can be explored.

**Figure 3:**
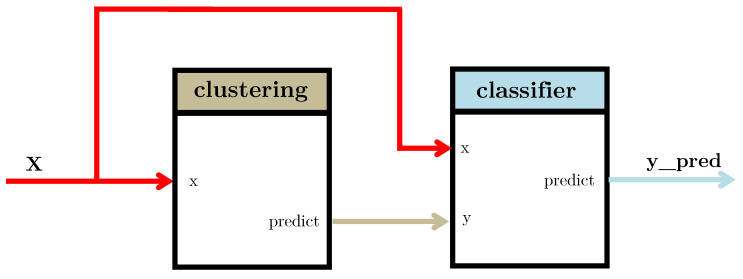
A pipegraph sporting two sequential model blocks.

For that purpose, a pipegraph model as the one shown in Figure 3 allows for a two model workflow. In this case, a clustering algorithm partitions a dataset consisting of 10,000 artificially generated 5-dimensional samples. It is worth noting that the partition is performed considering a specific and predefined number of clusters. This experiment splits the dataset in 5 folds for cross–validation. For each configuration, the classifier is trained and the quality of its results is assessed according to some convenient metric.The classifier considered was the well–known *k*-means training for a maximum of 300 iterations and a stopping criterion of error tolerance smaller than 0.0001.

**Figure 4:**
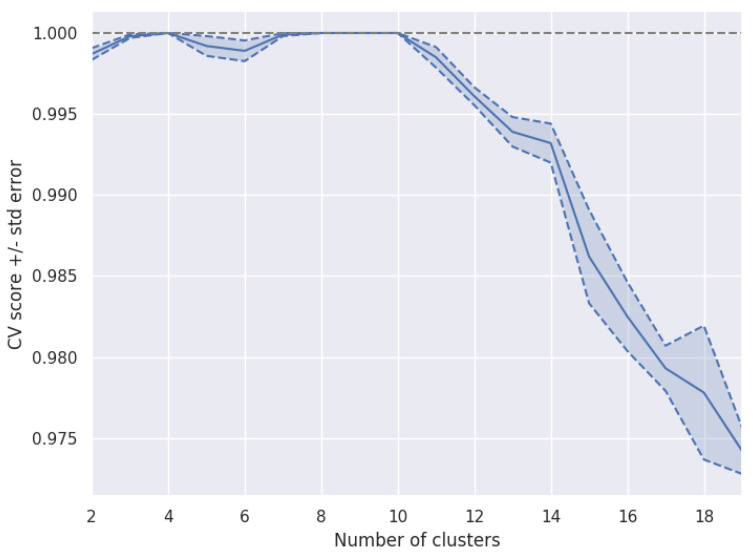
Number of clusters vs. a quality measure to assess the most appropriate number of clusters.

Figure 4 displays the quality results for such workflow in an illustrative case, described in more detail in the library documentation. It is worth noting that such a workflow cannot be constructed via standard scikit-learn tools as the two steps of the workflow are models and the standard Pipeline class only allows for a set of transformers and a single unique model in final position. We claim that for those purposes where different models are useful, even in a linear sequence, PipeGraph provides a viable and convenient approach.

### 4.2. Heat Exchanger Modeling

**Figure 5:**
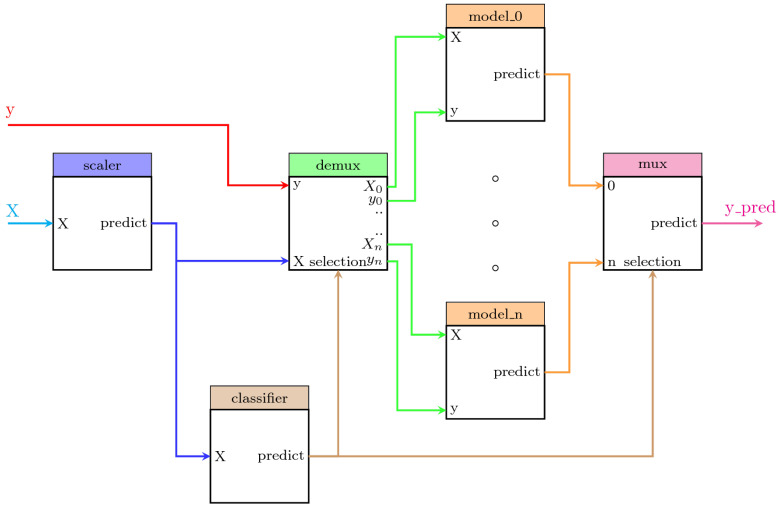
Parallel workflow with automatic wiring of the prediction models according to the labels contained in a dataset.

The second case study deals with a problem that is common in manufacturing processes: the management of faulty sensors [30]. In that paper, 52,699 measurements from a sensor embedded in a heat exchanger system were compared to the predictions from a baseline model capturing the expected behavior of such CPS system. If the sensor measurements were significantly different from the predictions provided by the CPS model, an alarm was raised and specific actions performed according to the particular case study. In the paper, two classes were again considered, namely day and night, standing for the two particular periods in which the 24 h are split. The best model obtained from a Cross-Validation setup using 10 folds was an Extremely Randomized Tree whose training explored a range from 10 through 100 base estimators. A pipegraph similar to the one considered in Figure 2 was used in the experience reported in the paper for a two model for two classes case. For such scenario, an approach using more than two classes could have been considered to check if such an enhanced model can outperform the results reported. Figure 5 displays a unified workflow in which PipeGraph is capable of automatically wiring as many local models as classes are defined in the dataset, thus relieving the user from the task of defining multiple configurations depending on how many classes were considered. It is again worth noting that such a workflow cannot be constructed via standard scikit-learn tools for the non linear workflow necessary for the purpose, and because of the automatic building procedure of the multiple prediction models put to play. We claim again that for those purposes where different models are used in a non linear sequence, PipeGraph provides a viable and convenient approach.

## 5. Conclusions

CPS and LM can greatly leverage on improvements in the tools and techniques available for system modeling. Data leakage, being one of the most common sources of unexpected behavior when the fitted models are stressed with actual demands, can largely be prevented by using encapsulation techniques such as the Pipeline provided by the scikit-learn library. For some specific complex problem appearing in the context of LM, and particularly in CPS modeling, the PipeGraph library provides a solution to building complex workflows, which is specially important for those cases that the standard Pipeline provided by scikit-learn cannot handle. Parallel blocks in non linear graph are available to unleash the creativity of the data scientist in the pursue of a simple and yet efficient model. In this paper we briefly introduced a novel PipeGraph toolbox for easily expressing ML models using DAG while being compatible with scikit-learn. We showed the potential of the toolbox and the underlying implementation details. We provided references to two case studies with hints on approaches for possible improvements by using PipeGraph and minor changes to the architecture proposed in two papers in the field of CPS and LM modeling. Future works on PipeGraph will include a Graphical User Interface (GUI) to use the API and new libraries which will encompass custom blocks related to specific areas connected to machine learning, such as computer vision, control systems, etc.

## Data Availability

The source code is available online at https://github.com/mcasl/PipeGraph (accessed on 20 December 2021), and the API documentation is available at https://mcasl.github.io/PipeGraph/api.html (accessed on 20 December 2021).

## Appendix A

**Listing A1.** Example code for the PipeGraph shown in Figure 2.
**import** numpy as np **import** pandas as pd **from** sklearn . preprocessing **import** MinMaxScaler **from** sklearn . linear_model **import** LinearRegression **from** sklearn . model_selection **import** GridSearchCV **from** pipegraph . base **import** PipeGraphRegressor **from** pipegraph . base **import** Demultiplexer **from** pipegraph . base **import** Multiplexer **import** matplotlib . pyplot as plt **from** sklearn . mixture **import** GaussianMixture X_first = pd . Series (np . random . rand (100,)) y_first = pd . Series (4 * X_first + 0.5 * np . random . randn (100,)) X_second = pd . Series (np . random . rand (100,) + 3) y_second = pd . Series (−4 * X_second + 0.5 * np . random . randn (100,)) X_third = pd . Series (np . random . rand (100,) + 6) y_third = pd . Series(2 * X_third + 0.5 * np . random . randn (100,)) X = pd . concat ([X_first, X_second, X_third], axis = 0) . to_frame () y = pd . concat ([y_first, y_second, y_third], axis = 0) . to_frame () scaler = MinMaxScaler () gaussian_mixture = GaussianMixture (n_components = 3) demux = Demultiplexer () lm_0 = LinearRegression () lm_1 = LinearRegression () lm_2 = LinearRegression () mux = Multiplexer () steps = [(’scaler’, scaler), (’classifier’, gaussian_mixture), (’demux’, demux), (’lm_0’, lm_0), (’lm_1’, lm_1), (’lm_2’, lm_2), (’mux’, mux), ] connections = { ’scaler’: {’X’: ’X’}, ’classifier’: {’X’: ’scaler’}, ’demux’: {’X’: ’scaler’, ’y’: ’y’,... ’selection’: ’classifier’}, ’lm_0’: {’X’: (’demux’, ’X_0’), ’y’: (’demux’, ’y_0’)}, ’lm_1’: {’X’: (’demux’, ’X_1’), ’y’: (’demux’, ’y_1’)}, ’lm_2’: {’X’: (’demux’, ’X_2’), ’y’: (’demux’, ’y_2’)}, ’mux’: {’0’: ’lm_0’, ’1’: ’lm_1’, ’2’: ’lm_2’,... ’selection’: ’classifier’}, } pgraph = PipeGraphRegressor (steps=steps,... fit_connections=connections) pgraph . fit (X, y) y_pred = pgraph . predict (X) plt . scatter (X, y) plt . scatter (X, y_pred)
